# Clinical heterogeneity among people with high functioning autism spectrum conditions: evidence favouring a continuous severity gradient

**DOI:** 10.1186/1744-9081-4-11

**Published:** 2008-02-20

**Authors:** Howard Ring, Marc Woodbury-Smith, Peter Watson, Sally Wheelwright, Simon Baron-Cohen

**Affiliations:** 1 Autism Research Centre, University of Cambridge, Cambridge, UK.; 2 Section of Developmental Psychiatry, University of Cambridge, Cambridge, UK.; 3 MRC Cognition and Brain Science Unit, University of Cambridge, Cambridge, UK.

## Abstract

**Background:**

Autism Spectrum Conditions (ASCs) are characterized by a high degree of clinical heterogeneity, but the extent to which this variation represents a severity gradient versus discrete phenotypes is unclear. This issue has complicated genetic studies seeking to investigate the genetic basis of the high hereditability observed clinically in those with an ASC. The aim of this study was to examine the possible clustering of symptoms associated with ASCs to determine whether the observed distribution of symptom type and severity supported either a severity or a symptom subgroup model to account for the phenotypic variation observed within the ASCs.

**Methods:**

We investigated the responses of a group of adults with higher functioning ASCs on the fifty clinical features examined in the Autism Spectrum Quotient, a screening questionnaire used in the diagnosis of higher functioning ASCs. In contrast to previous studies we have used this instrument with no *a priori *assumptions about any underlying factor structure of constituent items. The responses obtained were analyzed using complete linkage hierarchical cluster analysis. For the members of each cluster identified the mean score on each Autism Spectrum Quotient question was calculated.

**Results:**

Autism Spectrum Quotient responses from a total of 333 individuals between the ages of 16.6 and 78.0 years were entered into the hierarchical cluster analysis. The four cluster solution was the one that generated the largest number of clusters that did not also include very small cluster sizes, defined as a membership comprising 10 individuals or fewer. Examination of these clusters demonstrated that they varied in total Autism Spectrum Quotient but that the profiles across the symptoms comprising the Autism Spectrum Quotient did not differ independently of this severity factor.

**Conclusion:**

These results are consistent with a unitary spectrum model, suggesting that the clinical heterogeneity observed in those with an autistic spectrum condition at the higher-IQ end of the spectrum is associated with a gradient in the overall severity of the ASC rather than with the presence of different specific symptom profiles in different individuals. The implications of this for genetic research are considered.

## Background

Autism and the related disorders Asperger Syndrome (AS) and Pervasive Developmental Disorder Not Otherwise Specified (PDDNOS) are characterised by qualitative impairments of social interaction and communication, and restricted and repetitive patterns of behaviour of early childhood onset. They are now widely accepted as forming a spectrum of disorders, the so-called autism spectrum of conditions (ASCs), with the term 'spectrum' indicating the variation in the clinical phenotype observed [1]. Twin and family studies have demonstrated the etiological role of a number of genes [2-4], and linkage and association studies have begun to converge on regions of susceptibility and possible candidate genes respectively (reviewed in [5])

Unfortunately, however, the genetic research has been plagued with a failure to replicate findings, and with regions of linkage seemingly occurring on the majority of chromosomes. Recent research has taken two different approaches to address this. First, pooling data to increase sample sizes, such as with the Autism Genetic Resource Exchange [6] database, and secondly, examining subgroups within the autism spectrum, such as language delayed groups, those exhibiting repetitive patterns of behaviour and those meeting the more specific criteria for AS.

This latter approach is predicated on a model in which the spectrum is not a unitary concept, but, instead, consists of subgroups with unique but overlapping genetic influences [7,8]. At its most basic level this model argues that different sets of genes are responsible for the social, communication and behavioural impairments respectively. In support of this, the twin study of Ronald et al. 2006 [8] identified low phenotypic correlations between the three subscales of social interaction, communication and restricted/repetitive patterns of behaviour. There is also some genetic evidence to support this model. Shao [9], demonstrated improved linkage signals on chromosome 15q for a 'restricted and repetitive' behavioural subgroup, consistent with genes contributing differentially to these phenotypic characteristics. Although the genetic studies of Bradford et al. [10] and Buxbaum et al. [11] appear to also support this model, by considering language delay these studies have simply stratified their samples according to a general cognitive characteristic.

In contrast, however, other researchers have argued for a severity gradient [12,13], with presentation being modified according to the severity of autism symptoms. However, even if a severity gradient is the correct model it is still unclear whether this represents variation in severity of the core phenotype *per se*, or is a reflection of another factor, such as the impact of cognitive function on the expression of the phenotype.

There are a number of considerations that might explain the discrepancies between studies. An important consideration is the design of the studies. For example, Spiker's and Silverman's examined clustering according to the Autism Diagnostic Interview (ADI-R), whilst Constantino's [13] examined clustering on the Social Reciprocity Scale (SRS) and Ronald's [8] examined multivariate model fitting according to Childhood Asperger Syndrome Test (CAST). Clearly all questionnaires differ, as some are designed as screening instruments whilst others are aimed at diagnosis, some are concerned with higher functioning individuals whilst others are interested in a wider range of disability and some are self-report whilst others are informant-based. Other differences between studies included whether multiplex families or singletons were investigated, the sample sizes and the cognitive range of subjects included. All of these factors may have influenced the results.

In this current study, adult singletons with higher functioning ASCs are examined for their clustering on the fifty clinical features examined in the Autism Spectrum Quotient [14], a screening questionnaire used in the diagnosis of higher functioning ASCs. In contrast to previous studies we have used the AQ and have made no *a priori *assumptions about the underlying factor structure of this questionnaire and the clinical features it identifies. Our aim was to examine the possible clustering of responses within our study population and to determine whether the data obtained supported either a severity or a subgroup model to account for the phenotypic variation observed within the ASCs. Previous studies have sampled children with ASCs, but this is the first to examine an adult sample in this way. There is evidence that for a significant minority the ASC symptoms improve with age [15], suggesting that there may be different subgroups within this population, and raising the possibility that a different pattern of clustering will be observed among the adult ASC population. In addition, by including only those with higher functioning ASCs the effects of cognitive impairment *per se *on clustering are relatively reduced.

## Methods

The individuals comprising the study population were drawn from across the UK and had either been referred to a specialist diagnostic clinic (CLASS, or Cambridge Lifespan Asperger Syndrome Service) for clarification of a possible diagnosis of Asperger's syndrome or high functioning autism or had contacted the University of Cambridge Autism Research Centre (ARC) volunteer database. All participating individuals were aged at least 16 years and met diagnostic criteria for an ASC according to DSM-IV. Altogether 213 individuals were ascertained from the ARC volunteer database and 175 from the diagnostic clinic (CLASS), making a total of 388.

The characteristics entered into this study were those endorsed by each of the participants on the Autism Spectrum Quotient [14]. The AQ comprises 50 questions, covering 5 domains each of 10 questions assessing areas of relevance for the diagnosis of an autistic spectrum disorder, these being social skills, attention switching, attention to detail, communication and imagination. Each question is rated as 'definitely agree', 'slightly agree', 'slightly disagree', and 'definitely disagree', with approximately half the items worded to produce a 'disagree' response, and half an 'agree' response in a high scoring person with AS. The test-retest and inter-rater reliabilities of the AQ have also been shown to be good (*ibid*.). Moreover, the AQ has been found to be strongly predictive of a clinical diagnosis of AS according to DSM-IV criteria [16]; and has been shown to work well cross-culturally [17]).

For the purpose of this study, for each item a score of 0,1,2 or 3 was assigned along the spectrum 'definitely agree', 'slightly agree', 'slightly disagree', and 'definitely disagree' for items worded to produce a 'disagree' response in a high scoring person with AS, and 3,2,1 and 0 for items worded to produce an 'agree' response. Therefore, each individual's scores ranged from 0 to 3 for each of the 50 AQ items, and 0 to 150 for the complete questionnaire, with higher scores indicative of traits more typical of ASCs. Subsequently, complete linkage cluster analysis was employed [18], using SPSS Version 11 [19].

## Results

AQ responses from a total of 388 individuals between the ages of 16.6 and 78.0 years were initially considered. Of these, 127 were female and 261 were male. There was no difference in mean AQ score between those recruited from the ARC volunteer database and those seen in the CLASS clinic (ARC = 37.3, Clinic = 36.8: t = .67, P = 0.502). Mean ages (ARC = 35.3 years, Clinic = 32.6 years) in the two populations were similar. Considering gender distribution (ARC = male 57%, Clinic = male 79%, Fisher's exact test P <0.001), the preponderance of males with respect to females attending the diagnostic clinic was significantly greater than was the case for members of the ARC volunteer data base. Although IQs were unfortunately not available, all participants had attended mainstream school. In 55 participants some AQ responses were missing and these individuals were excluded, leaving questionnaires from 333 individuals to be included in the cluster analysis.

**Figure 1:**
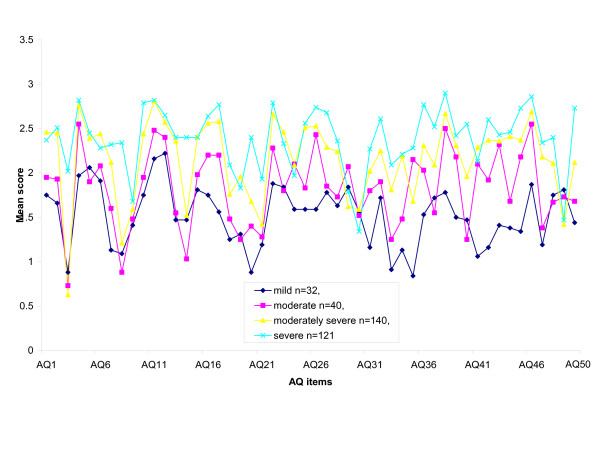
Group mean scores for each of the fifty AQ items.

Whilst the 2, 3 and 4 cluster solutions produced groups each with more than 10 participants, in contrast, solutions with more than four clusters included one or more groups with very small numbers (i.e. with less than 10). The significance of groups with very small numbers such as these is unclear, and therefore only the 2, 3 and 4 cluster solutions were examined further. For each AQ item, the group mean was calculated, allowing comparison between groups for each of the 50 AQ items. By way of illustration, Table 1 shows the group mean scores for the 4 cluster solution, shown graphically in Figure 1.

**Table 1:** Mean Cluster scores for each AQ item in the 4 cluster solution

	'mild' N = 32	'moderate' N = 40	'moderately severe' N = 140	'severe' N = 121
AQ1	1.75	1.95	2.46	2.37
AQ2	1.66	1.93	2.45	2.51
AQ3	0.88	0.73	0.63	2.02
AQ4	1.97	2.55	2.76	2.82
AQ5	2.06	1.9	2.39	2.45
AQ6	1.91	2.08	2.44	2.28
AQ7	1.13	1.6	2.12	2.32
AQ8	1.09	0.88	1.21	2.34
AQ9	1.41	1.48	1.59	1.68
AQ10	1.75	1.95	2.44	2.79
AQ11	2.16	2.48	2.81	2.82
AQ12	2.22	2.4	2.57	2.65
AQ13	1.47	1.55	2.36	2.4
AQ14	1.47	1.03	1.52	2.4
AQ15	1.81	1.98	2.41	2.4
AQ16	1.75	2.2	2.56	2.64
AQ17	1.56	2.2	2.58	2.77
AQ18	1.25	1.48	1.76	2.09
AQ19	1.31	1.25	1.96	1.83
AQ20	0.88	1.4	1.68	2.4
AQ21	1.19	1.28	1.42	1.93
AQ22	1.88	2.28	2.66	2.79
AQ23	1.84	1.8	2.46	2.33
AQ24	1.59	2.1	2.09	1.97
AQ25	1.59	1.83	2.51	2.56
AQ26	1.59	2.43	2.53	2.74
AQ27	1.78	1.85	2.29	2.68
AQ28	1.63	1.73	2.24	2.36
AQ29	1.84	2.07	1.62	1.79
AQ30	1.56	1.52	1.59	1.34
AQ31	1.16	1.8	2.02	2.27
AQ32	1.72	1.9	2.25	2.61
AQ33	0.91	1.25	1.81	2.09
AQ34	1.13	1.48	2.19	2.21
AQ35	0.84	2.15	1.68	2.28
AQ36	1.53	2.03	2.31	2.77
AQ37	1.72	1.55	2.09	2.52
AQ38	1.78	2.5	2.67	2.9
AQ39	1.5	2.18	2.31	2.42
AQ40	1.47	1.25	1.96	2.55
AQ41	1.06	2.1	2.29	2.13
AQ42	1.16	1.92	2.37	2.6
AQ43	1.41	2.32	2.36	2.43
AQ44	1.38	1.68	2.41	2.46
AQ45	1.34	2.18	2.37	2.73
AQ46	1.87	2.55	2.69	2.86
AQ47	1.19	1.38	2.18	2.34
AQ48	1.75	1.67	2.11	2.4
AQ49	1.81	1.73	1.42	1.47
AQ50	1.44	1.68	2.12	2.73

## Discussion

Strikingly, each of these solutions was consistent with a severity gradient. Considering the 4 cluster model for example, although not all AQ items demonstrate a clear severity gradient, 64% of the AQ items are consistent with gradient of severity with Group 1 representing 'severe' cases, Group 2 'moderately severe', Group 3 'moderate' and Group 4 'mild'. Similar results were found for the 2 and 3 cluster models.

In summary, the results of this current analysis support a unitary spectrum model, with clustering according to severity. This study therefore seems primarily to agree with that of Spiker and colleagues [12], who have argued for a continuous severity gradient. In contrast, the results did not provide support for Silverman et al. [7], who have argued for subgroups.

There are several limitations to the current study that should also be considered. The lack of IQ data precluded us from examining the impact of cognitive functioning on AQ scores. The spread of ages within the participant population, though excluding children below the age of 16, was large. The smaller male excess in the ARC sample with respect to the CLASS Clinic sample was unexpected. The sex ratio in the clinic participants resembled that recorded in many other studies of people with ASC. The clinic population were referred by other doctors (family doctors, psychiatric services) who may have been influenced in their willingness to refer to a specialist service by expectations that autism-like symptoms when seen in males may be autism but when seen in females may have some non-autistic cause. The observation that both the ARC-ascertained and the clinic-ascertained samples had similar age profiles and similar scores on the AQ suggests that people with conditions of similar severity presented to both.

Although these various factors may have influenced the results, it remains possible that a gradient of severity is an independent observation and explains the variation between people's clinical phenotype. Such a gradient may not necessarily be the result of differences in autism symptoms per se, but might instead reflect the modifying effect of another continuously distributed factor (or factors). For example, IQ is continuously distributed in the population and differences in IQ between participants may have mediated the gradient observed. Unfortunately, IQ scores were not available for the participants of this study, and so to examine this hypothesis further data are needed. Similarly, personality traits vary quite considerably in the general population and these may have a modifying effect on the expression of the phenotype. This too needs further investigation. However, the variation in severity observed in this and previous studies may be due to a variation in severity of autism itself, and, if so, this may be the result of an additive genetic effect for autism related genes. This model proposes that there are several susceptibility genes for autism, and that the more of these genes a person has, the more severe will be the phenotype. These genes may be conceptualised as conferring a general susceptibility to autism rather than comprising different genes that contribute to different aspects of the phenotype such as social interaction, communication, rigidity and so forth. The results of the current study and previous ones [12,13] support this model. If a genetic dosage effect does, indeed, explain phenotypic variation, then the first degree relatives would show a similar trend of severity for autism traits. Unfortunately, data is not available from family members for this hypothesis to be tested in the current study.

These analyses should be considered as exploratory. The ideas developed in this paper need to be tested further for replication, and examined across a wider spectrum of disability. Clearly what is now needed is the exploration of factors that might contribute to the existence of a severity gradient, and more specifically whether this can best be explained by the modifying effect of another continuously distributed factor, such as IQ, or whether it is indicative of ASCs being inherited as a single, continuous severity dimension. Ultimately, examination of inter versus intrafamilial clustering, both in affected sib-pairs, and within their unaffected parents, will be able to investigate the hypothesis that the severity gradient is the result of the modifying effect of another non-ASC factor, such as IQ. Until this is clearer, difficulties in identifying ASC susceptibility genes may continue.

## Conclusion

The clinical heterogeneity observed in people with ASCs, together with the strong hereditability recognised in these conditions, has led to a debate as to whether this variation in manifestation is associated with either distinct genetic sub-types with quite different clinical symptoms or with a common basis but with clinical variability originating as a result of an overall variation in severity between individuals. Examination of the responses of over 300 individuals with Asperger's syndrome or high functioning autism to the 50 items of a questionnaire rating the occurrence of a wide range of behaviors characteristic of ASC provides evidence that the observed clinical spectrum is associated with a gradient in the overall severity of the ASC rather than with the presence of different specific symptom profiles in different individuals. The possible causes for this variation in severity however remain to be established.

## Abbreviations

CLASS: Cambridge lifespan asperger syndrome service; AQ : Autism spectrum quotient questionnaire; ARC : University of cambridge autism research centre; ASCs : Autism spectrum conditions

## Competing interests

None of the authors have any competing financial interests. The AQ was developed by Sally Wheelwright and Simon Baron-Cohen but it is freely available and they do not derive any financial benefit from its use.

## Authors' contributions

The study was devised by HR and MWS. SW and SBC prepared the data for analysis and advised on interpretation of the results. PW carried out the statistical analysis. HR and MWS wrote the first draft of the paper and all the authors contributed to modifications in subsequent drafts.
